# A Vision for Development and Utilization of High-Throughput Phenotyping and Big Data Analytics in Livestock

**DOI:** 10.3389/fgene.2019.01197

**Published:** 2019-12-17

**Authors:** James E. Koltes, John B. Cole, Roxanne Clemmens, Ryan N. Dilger, Luke M. Kramer, Joan K. Lunney, Molly E. McCue, Stephanie D. McKay, Raluca G. Mateescu, Brenda M. Murdoch, Ryan Reuter, Caird E. Rexroad, Guilherme J. M. Rosa, Nick V. L. Serão, Stephen N. White, M. Jennifer Woodward-Greene, Millie Worku, Hongwei Zhang, James M. Reecy

**Affiliations:** ^1^Department of Animal Science, College of Agriculture and Life Sciences, Iowa State University, Ames, IA, United States; ^2^Animal Genomics and Improvement Laboratory, USDA-ARS, Beltsville, MD, United States; ^3^College of Agriculture and Life Sciences, Iowa State University, Ames, IA, United States; ^4^Department of Animal Sciences, University of Illinois at Urbana-Champaign, Urbana, IL, United States; ^5^Animal Parasitic Diseases Laboratory, United States Department of Agriculture, Agricultural Research Service, Beltsville, MD, United States; ^6^Department of Veterinary Population Medicine, College of Veterinary Medicine, University of Minnesota, Saint Paul, MN, United States; ^7^Department of Animal and Veterinary Sciences, College of Agriculture and Life Sciences, University of Vermont, Burlington, VT, United States; ^8^Department of Animal Sciences, University of Florida, Gainesville, FL, United States; ^9^Department of Animal and Veterinary Science, University of Idaho, Moscow, ID, United States; ^10^Department of Animal and Food Sciences, College of Agricultural Sciences and Natural Resources, Oklahoma State University, Stillwater, OK, United States; ^11^Agricultural Research Service, United States Department of Agriculture, Washington D.C., DC, United States; ^12^Department of Dairy Science, University of Wisconsin-Madison, Madison, WI, United States; ^13^Animal Disease Research Unit, Agricultural Research Service, United States Department of Agriculture, Pullman, WA, United States; ^14^Department of Veterinary Microbiology and Pathology, College of Veterinary Medicine, Washington State University, Pullman, WA, United States; ^15^Center for Reproductive Biology, College of Veterinary Medicine, Washington State University, Pullman, WA, United States; ^16^Department of Animal Sciences, North Carolina Agricultural and Technical State University, Greensboro, NC, United States; ^17^Department of Electrical and Computer Engineering, College of Engineering, Iowa State University, Ames, IA, United States

**Keywords:** automated phenotyping, precision agriculture, precision livestock farming, phenomics, sensors

## Abstract

Automated high-throughput phenotyping with sensors, imaging, and other on-farm technologies has resulted in a flood of data that are largely under-utilized. Drastic cost reductions in sequencing and other omics technology have also facilitated the ability for deep phenotyping of livestock at the molecular level. These advances have brought the animal sciences to a cross-roads in data science where increased training is needed to manage, record, and analyze data to generate knowledge and advances in Agriscience related disciplines. This paper describes the opportunities and challenges in using high-throughput phenotyping, “big data,” analytics, and related technologies in the livestock industry based on discussions at the Livestock High-Throughput Phenotyping and Big Data Analytics meeting, held in November 2017 (see: https://www.animalgenome.org/bioinfo/community/workshops/2017/). Critical needs for investments in infrastructure for people (e.g., “big data” training), data (e.g., data transfer, management, and analytics), and technology (e.g., development of low cost sensors) were defined by this group. Though some subgroups of animal science have extensive experience in predictive modeling, cross-training in computer science, statistics, and related disciplines are needed to use big data for diverse applications in the field. Extensive opportunities exist for public and private entities to harness big data to develop valuable research knowledge and products to the benefit of society under the increased demands for food in a rapidly growing population.

## Introduction

### Why Develop High-Throughput Phenotyping Systems in Livestock?

The hope and excitement around the use of “big data” in agriculture hinges on the potential to harness information to produce food in a more sustainable way while meeting the nutritional demands of a growing world population. The term “big data” has been used to describe new information collected by automated or high-throughput systems, which has been commonplace in some sectors of the livestock industry (i.e., milk recording sector of the dairy industry) for more than 40 years. However, these data have been largely under-utilized in the past, and integration of information sources by segments of the livestock industry has been poor or non-existent. The livestock industry is rapidly adopting precision agriculture technologies in the form of wearable sensors in all species to improve animal efficiency, health, and welfare. However, there are still many types of data that are not being used for analytics or predictive ability, which could further enhance the sustainability of livestock industries (e.g., in the US: feed composition information, methane emission measures, mid-infrared milk spectral data, and real-time digital images). Historically, the ability to collect and utilize on-farm data for breeding purposes revolutionized the beef, dairy, poultry, and swine industries, leading to huge gains in productivity and efficiency (Hill, 2016). Similar opportunities for tremendous gains may be possible with the utilization of sensors and other high-throughput phenotyping technologies in many other contexts of the livestock industry (Science Breakthroughs to Advance Food and Agricultural Research by 2030, 2019).

A number of publications have highlighted the initial applications and potential of big data and technologies to transform the animal sciences. Several reviews of precision livestock farming (PLF) present opportunities and initial examples of how PLF has been used in animal agriculture (Berckmans, 2017; Guarino et al., 2017; Wolfert et al., 2017). Notable examples include the ability to detect animal health problems requiring human intervention (e.g., lameness in cattle), monitor animal behavior to evaluate the function of feeding systems that impact health of broiler chickens, and the use of sounds to detect illness (Berckmans, 2017). Digital agriculture and PLF have been touted as a means to improve precision of breeding, feeding, and management of animals and plants in a way that will improve sustainability and reduce the environmental footprint associated with agriculture (Weersink et al., 2018). Efforts in Europe to advance PLF technologies and applications have largely been more advanced than those in the United States. This has led to the development of more advanced research and conceptual models demonstrating how PLF may lead to “smart farming” (i.e. automated and real-time decision making) where a clear solution exists to improve animal health, productivity, and well-being (Guarino et al., 2017; Wolfert et al., 2017). Recently, two groups of animal scientists have presented a perspective of how precision livestock technologies may be used to mine knowledge from data and facilitate the development of new technologies though academic and industry collaborations. Morota et al. (2018) describe how animal science can be transformed into a data science with the rich quantity of digital data and new machine learning methods available to glean information from these data. Ramirez et al. (2019) provide a perspective of how industry and stakeholders perceive the challenges and opportunities of utilizing precision technologies in livestock, with considerable discussion on data ownership, impact on rural economic development, and how and where value may be realized from PLF data. A common theme among all of these manuscripts is the need to determine how to make better use of existing data, share data across industry and academia, and address the need for training in academia to catch up with advances made by industry.

### What Are the Grand Challenges That Frame Our Values for Making Use of “Big Data”?

All sectors of agriculture will need to more than double their productivity to feed a growing population that is estimated to exceed 10 billion people by 2050 (Food and Agriculture Organization of the United Nations (FAO), 2018). Livestock-derived foods fill a unique and important role in that they can provide essential vitamins and minerals not available through plant sources, and which are generally poorly absorbable from synthetic sources. In addition, livestock are an excellent protein source and some animals, such as ruminants and fish, can make use of marginal lands and seas that would otherwise be difficult to cultivate. In order to meet the needs of the growing human population, the livestock community realizes that there are challenges and opportunities to be more efficient, environmentally friendly, and attentive to societal needs. These challenges provide opportunities to develop more sustainable and profitable agribusiness. The types of data needed to meet these grand challenges are diverse and present many opportunities for scientific discovery to link genotype to phenotype, develop computational tools for big data analytics, engineer new sensor technologies, develop new systems to coordinate data, and ultimately utilize this information for improved animal production and welfare.

### What Are Big Data?

While the term “big data” has become ubiquitous, its meaning is often unclear, particularly because there is a tendency to conflate the data with analytical methods applied to those data. The so-called four V model (IBM, http://www.ibmbigdatahub.com/infographic/four-vs-big-data) defines big data based on properties of the collection, but a survey of the literature shows that the Vs are growing as quickly as data themselves, from 4- to 7- to 10- to 42-V models. The four V model defines big data based on four key attributes: 1) volume, 2) velocity, 3) variety, and 4) veracity. Volume is simply the quantity of data available. Velocity is the speed at which users want to access or use the data. Variety describes the different forms in which the data are received. Veracity focuses on the need to clean and edit large amounts of data so that sound inference can be drawn from quality-controlled records. Value is commonly added to V models because, as data become cheaper to collect, the utility of those observations are often suspect, unless there is an improvement in the methods/technology used to generate the data and subsequent quality of these data. However, it is not clear that this needs to be explicit in the livestock sector because the value proposition is at the heart of the decision to collect data in the first place. Validation or verification of quality is more important as the use of “big data” will rely on careful data editing to remove noise and focus on the informative aspects of the data that are valuable for analytics.

### What Is Big Data in a Livestock Context?

While data have been critical to process evaluation and decision-making in almost all modern business and scientific enterprises, the modern era of “big data” is closely tied to the development of new technologies that permit the inexpensive, rapid collection of many observations. The Human Genome Project (Green et al., 2015) and the Large Hadron Collider at the European Organization for Nuclear Research (ACM, 2011) are canonical examples of big data in the sciences. In the general livestock community, a symposium on big data titled “Really Big Data: Processing and Analysis of Very Large Data Sets” (Cole et al., 2012) was held at the 2011 Joint Annual Meeting of the American Dairy Science Association (ADSA) and the American Society for Animal Science in New Orleans, Louisiana. This was followed in 2016 by the 31^st^ ADSA Discover Conference^SM^ on Food Animal Agriculture, which had the theme of “Big Data Dairy Management.” The subject has even drawn Congressional attention, with a House Committee on Agriculture hearing on private big data in agriculture (Stubbs, 2016). This interest was driven by the development of large genomic datasets (Daetwyler et al., 2014), as well as the development of on-farm sensors that can provide continuous, real-time observations of an animal’s environment and performance [e.g., Rutten et al. (2013)]. There are also rapidly growing collections of mid-infrared milk spectral data which show potential for use as indirect predictors of many traits that are expensive or difficult to measure directly (e.g., De Marchi et al., 2014; Dorea et al., 2018).

There is a growing literature on precision agriculture, which often overlaps with many big data concepts (e.g., Wolfert et al., 2017; Morota et al., 2018; Weersink et al., 2018). The goal of precision agriculture is to use detailed, frequently collected observations on individual animals to make appropriate management decisions. In a livestock setting, this includes identification of changes in productivity, determination of reproductive status, early detection of health problems at the individual and group levels, and grouping of animals with similar nutritional or other management needs. Movement from the barn or pen level to the farm or landscape level (i.e., country, state or regional location attributes) can be accompanied by dramatic growth in the data available, including soil composition, water availability and utilization, and weather information.

### What Does the Livestock Community Need to Capitalize on Opportunities Provided by High-Throughput Phenotyping Technologies and Other “Big Data”?

Sensor, image, and routine laboratory data are collected at many different times in an individual’s life in several livestock species. Companies are beginning to capitalize on these technologies by developing sensors, computer systems, and analytic tools to market information to livestock producers that can be used to prevent health problems, monitor animal welfare, and behavior, and improve fertility, efficiency, and profitability. There are still many unexplored opportunities to develop new monitoring technologies and much work remains to develop predictive analytics to utilize this data to its full potential. *The objective of this manuscript* is to define opportunities and gaps for the use of high-throughput phenotyping, “big data” analytics, and related technologies in the livestock industry. The information expressed herein is based on direct feedback and comments from participants of the Livestock High-Throughput Phenotyping and Big Data Analytics meeting held November 13 and 14, 2017 at the USDA National Agricultural Library in Beltsville, MD. This meeting included academics, industry representatives, and agents from various funding agencies to gain a wide perspective of opportunities for big data use in livestock. To make the most use of data and enhance further development in precision livestock technologies, investments will be needed in infrastructure for people (e.g., “big data” training), the data itself (e.g., data transfer), and technology (e.g., development of low cost sensors). These three pillars, which we will refer to as the data-driven decision triad, will be the basis for describing the gaps and opportunities to use “big data” technologies to advance livestock sustainability. Since the focus of these technologies is to gain more information about livestock, animals are central to these three focus areas. These challenges extend beyond livestock and have wide applicability within society to better use data to create advancements in society. These infrastructure investments can be thought of like bricks needed to construct a building (Platt, 1964).

## People

Understanding and training in agriculture and specific livestock areas will be critical to the development of real-world big data and high-throughput phenotyping applications. Training will be needed for individuals entering the workforce as well as those currently working in the livestock industry. Furthermore, interdisciplinary teams will be needed as no one scientist or field will be able to solve all challenges in this area. This may be especially challenging for junior graduates and scientists because of their limited experience across multiple disciplines. Further, there is a critical need to interact with those outside of science to discuss societal needs and impacts related to how these technologies will be translated into real-world applications. Attracting people with complementary skills from outside of agricultural fields, such as engineers, mathematicians, computer scientists, software developers, design specialists, and sociologists will be important to help solve data-driven problems. However, there are still limited professionals in the area of “big data.”

### Big-Data Faculty Positions

From 2015 to 2020, U.S. agriculture will create about 60,000 jobs every year requiring graduates with bachelor’s or higher degrees in food, agriculture, renewable natural resources, or the environment (Employment Opportunities for College Graduates, 2015). With such need for well-trained graduates, U.S. universities need to create faculty positions focused on “big data” in livestock production. These faculty members should have a deep understanding of animal production, data management, digital agriculture, and statistical methodologies, in order to properly train inside (i.e., through development of new courses) and outside (e.g., through research projects and extension activities) undergraduate and graduate students to fill these jobs. Departments related to crop sciences, agricultural engineering, data sciences/bioinformatics, and statistics, have opened several faculty positions in “big data” in the past years to fill this need. In animal science departments, however, this has not been the case. Creating “big data” faculty positions within departments of animal science across the U.S. will be an important step to take U.S. animal production to greater levels.

### Critical Training Needs for Students

Animal Science departments have recently begun to hire “big data” faculty members with expertise in computational biology, precision agriculture, and livestock appointments across the country. However, current students (undergraduate and graduate) still need more education in data science, including statistics, computer science, and related areas, to build the human capacity need for livestock big data research. Among our first priorities, students will need to understand the big picture opportunities to apply “big data” analytic skills within the animal science field. Internships with industry and interdisciplinary cross-training will help students to see application beyond the data science. With a dwindling number of students coming from agricultural backgrounds, recruiting young “urban” students into agriculture will be required to fill new job opportunities. Such students with backgrounds in computer science, data management, and other related areas need exposure to the exciting challenges in agriculture, and opportunities to give them experience with our big data needs are one way to connect such students with real-world agricultural applications, stimulate interest in this work, and improve broader societal awareness of animal agriculture. This can take the form of internships tailored to recruitment, faculty, or companies providing step-by-step examples online, existing faculty teaching courses across programs, existing faculty volunteering to serve as advisors, co-advisors, or rotational advisors for students from computer science and big data degree programs, or cross-training by establishing joint degree programs. Importantly, education about agricultural data sciences should start before college, in 4-H (https://4-h.org/) and elementary/high-school (e.g., in FFA programs; https://ffa.org/), so future workers learn about the great opportunities to work in this area. In addition, there is a need for big data, high-throughput phenotyping, informatics, and data analytics-based fellowships to recruit talented students. Good mentoring of these students who would be cross-trained in multi-disciplinary skillsets will be critical to keep students on track in their studies. Development of online learning programs across universities like the Great Plains IDEA (https://www.gpidea.org/program/animal-science) approach would be good to facilitate wide dissemination of training in these areas.

### Importance in Training the Existing Research Workforce

This training should be applied not only to new students, but also to established scientists to bring all researchers up-to-date such that there is a shared language and understanding of important terms for the development of “big data” analytical tools and technologies. Instruction within the animal sciences is needed in database design, data management, general programming, and increasingly in statistical inference to work with the varied types, and volume of data being generated by new automated phenotyping systems. This may facilitate the need for data scientists to provide “research extension” to train the existing workforce on big data analysis methods. Online courses are already available for general concepts of big data (e.g., big data Hadoop; https://intellipaat.com/big-data-hadoop-training/), but nothing on agriculture. Successful development of such tools will help train the existing research workforce, and can be transferred to outside groups like computer scientists and big data degree programs in a virtuous cycle to improve recruitment to agricultural settings. Furthermore, workshops and community-driven efforts to continually update the existing research workforce on new data analytics methods are needed to promote creative development of new research in big data fields. With the generation of trained graduates in livestock big data, training of the current workforce will soon be reality.

## Technology

The ability to harness large, diverse data for precision livestock farming requires considerable development of automated phenotyping technologies, e.g. development of new sensors that can dramatically decrease the cost of collecting data that is currently very expensive to collect. Further, both statistics for data analytics and computation in the form of hardware and software must be improved. Standardization and replicability of data from different technologies is also important to facilitate data reuse and integration. Additionally, the ability to transfer large volumes of data across networks in real-time is a challenge and opportunity with the technologies needed for big data analytics. Development of improved broadband, data compression, and data transfer methods are needed to get data from the field to analytical tools, especially when data originate in rural areas. Interdisciplinary teams will be needed to facilitate the development of technology and implement its use in a meaningful way to allow data to be used for a variety of management purposes.

### Rural Broadband

Today, most livestock farms (especially remote, open farms) are not connected at all, and 39% of the rural U.S. lacks broadband access (Zegura et al., 2017). To enable high-throughput field phenotyping, we need to develop high-throughput, low-cost rural broadband solutions, and the solutions need to support the wide range of phenotyping needs. For instance, there is a need to connect diverse, pervasive data types such as animal-worn bio-sensors and statically deployed, unmanned aerial vehicle (UAV)- mounted high-definition cameras with high-throughput wireless connectivity in animal barns and large, open-grazing fields. To this end, we need to effectively leverage the variety of wireless network architectures and technologies that may be suitable for rural deployment. We need to enable animal-worn devices to self-organize into low-rate sensor networks and leverage renewable energy to power wireless backbone networks covering large, open fields. To provide network coverage in all rural areas, there is a need to leverage emerging wireless technologies such as high-capacity, long-range millimeter-wave wireless backhauls, open-source wireless innovation platforms such as the universal software radio peripheral (USRP) software-defined radios, as well as the wireless spectrum in TV White Space and other frequency bands such as the citizens broadband radio service (CBRS) and industrial, scientific, and medical (ISM) bands. We need to develop and deploy wireless living labs so that future-generations of rural broadband solutions can be rolled out in the fastest and most effective manner. It is expected that many of the rural broadband networks will be owned and operated by local communities/farmers, thus challenging the traditional models of having large commercial internet service providers (ISPs)/carriers and offering the opportunity of developing farmer-technologists for sustainable rural development. There is also the opportunity of aligning rural broadband initiatives with various government initiatives, such as the USDA rural broadband programs (USDA Rural Development Innovation Center, 2019: https://www.rd.usda.gov/files/508_RDeConnectivityToolkit121918.pdf).

### Computation

The availability of adequate computational power and development of software will be critical to fully utilize serially collected high-throughput phenotypes. The ability to share existing computer power at national computing centers, and training on how to use this infrastructure, will be important in data analytics-based research. Data management, processing, and application will be critical to make use of big data for PLF. Software that can track, manage, and move data to and from databases rapidly will also be critical to keep all metadata and data points organized and connected. Software facilitating high-throughput extraction of large quantities of data such as MapReduce (https://en.wikipedia.org/wiki/MapReduce), Pig (https://pig.apache.org/), Hive (https://hive.apache.org/), and Hadoop systems (https://hadoop.apache.org/) has been developed. However, the true challenge is in creating efficient database structures and facilitating functional data flow with diverse data types that allow flexibility in data query and downstream use. Development of machine learning, artificial intelligence based computing, or other methods will be important to rapidly turn data into knowledge that can be used for real-time management decisions on-farm. Development of computer vision is extremely promising for automated phenotyping using image data. In addition, software will need to be developed to allow for easy access to large databases so that data can be reused for different analyses, genetic evaluations, management evaluation, and overall evaluation of on-farm management. Software will need to be portable across systems as well as easy to deploy and access, for example, deployment in containers such as Docker (https://www.docker.com/) or Singularity (https://sylabs.io/singularity/), and accessible in repositories like Bit Bucket (https://bitbucket.org) and GitHub (https://github.com/). Specifically, Bit Bucket and GitHub facilitate sharing of both software and user documentation which can be instrumental in broader use and integration of new technologies. An exciting possibility is use of applications on cell phones both to collect data and interact with processing software to get information for real-time management decisions. Since cell phone hardware is already present in many animal care situations, cell phones have the potential to extend phenotype collection ease and convenience at low or no cost. With such a large amount of data that can be collected, data reduction and network speeds can play critical roles to mitigate potential bottlenecks to analyzing large datasets caused by limits in data storage and transfer rates.

The research community is embracing the challenges in computation and software development for precision livestock phenotyping tools. However, adoption in the field by producers, industry, and veterinary practitioners will mark the full realization of its potential. Effective user interface and data visualization tools to manage and interpret data from diffuse precision sensors in production settings will be essential for adoption. The use cases for non-research groups will require engaging and straightforward user interfaces available on varied devices. This approach will yield a comprehensive understanding of the factors impacting animal welfare and management, which in turn, can empower farmers to identify emerging issues and aid real-time decisions leading to increased production, efficiency, and animal health and comfort.

The ultimate dissemination and scalability of any new precision livestock tool may hinge on researcher’s initial design capacity for this type of multi-tool aggregation and data visualization, as well as consideration of the established factors leading to new technology adoption. If the end user tools are too complex, expensive, or impractical, they will not be adopted outside of research. Van Hertem et al. (2017) noted that “most farmers do not have the skills and time to utilize new precision livestock technologies effectively.” To enhance farmer acceptance of several precision sensor technologies, they developed a data visualization tool to enhance the user experience and adoption of the technologies. Pierpaoli et al. (2013) showed that farmer familiarity with computers was second only to farm size among several features affecting farmer acceptance of new agricultural technologies.

New tools such as ADOPT (Kuehne et al., 2017), and other models designed to predict potential adoption, may inform researchers and policy planners in designing and identifying potentially high impact precision agriculture research and development. Strategies to address these issues could be outlined in research data management plans, and provide a roadmap for how the new technology could be advanced beyond initial development. In addition to ensuring consideration of farmer adoption by the researchers in their design, the prospective roadmap to adoption could guide future efforts to include multi-tool integration, data visualization, and user interface development and training.

### Statistics for Data Analytics

A major challenge with large, serially collected data is how to assure that appropriate statistical models are used when hundreds or even millions of variables need to be included in thousands to millions of models. In these cases, it is challenging to select a single model that can best fit each response variable. Quality control with data standardization and calibration methods are needed to screen such large data sets to help remove noise from data that may inhibit analytics. Software and methods are needed to check and adjust each individual model for outliers, heterogeneous variance, and other statistical challenges. In addition, most automated technologies involve serial data collection from the same animal, over time, within similar environments, which introduces shared covariance that needs to be modeled to adjust for systematic effects on a given response variable. New statistical methods may need to be developed to better account for these various challenges; as longitudinal data collected across time is very valuable in determining animal health, growth, and efficiency phenotypes, while also taking full advantage of insights from multivariate models. Appropriate analytics will also be needed to summarize data. In addition, new visualization and data integration methods are needed to better view and use information within these large data sets. Development of new algorithms, analytical tools, and approaches utilizing these digital measurements will require phenotypic data that is accessible, and uniform in structure and format from the start of data collection. But, to meet the challenge and opportunity to understand and make use of phenotypic data in the twenty-first century, we need to think beyond phenotypes, or even the combination of genotype and phenotype alone (Rexroad et al., 2019).

### Data Integration

Big data algorithms and techniques are designed to analyze digital data gathered from many sources. The data may be massive in quantity, and are often irregular or non-uniform in data points, data format, data structure, or in some or all of these aspects. The data gathered must then be integrated for analyses, interpretation, and visualization. The promise of big data approaches is to provide insights through data integration that were not possible before this type of mass data collection was developed (McCue and McCoy, 2017). The complexity of interactions that regulate complex, measurable production, or health traits in farm animals is well-suited to big data applications and approaches. Development of database systems, statistical methods, and visualizations that facilitate data integration will be critical to realize this promise. The great challenge for success and, thus, an immediate need to utilize big data techniques fully, are for strategic planning for data management, including data security, storage capacity, accessibility, and integrity resulting in data of high quality—i.e., collected, well-documented, uniformly documented, and formatted across data sources (“Notes from: Genome to Phenome: A USDA Blueprint for Animal Production” 2017). Perhaps the greatest obstacle that will limit high-throughput phenotyping is not lack of the workforce or technology shortcomings, but the lack of needed legal framework to effectively assemble and utilize this information for improved livestock production. Ownership of data, at least under the US legal system, is not a clear-cut issue, particularly because data are non-rival goods (one person’s possession of the data does not deprive another of the ability to use the data, as might be the case for a tractor) (de Beer, 2016). This means that manufacturers of equipment, such as a milking system, may assert that they are the owners of data generated by that system, rather than the farmer from whose animals the observations were taken. Resolving such issues will take time, but these issues need to be resolved in order to realize the full potential of high-throughput phenotyping technologies.

### Automated Phenotyping Technologies

Automated phenotyping technologies need to collect, store, and/or transmit data recorded serially to a data receiver for further processing. Sensor and imaging data are particularly promising as they require no additional animal handling, which would drastically accelerate animal phenotyping while eliminating labor and animal stress. These technologies could monitor complex metabolic processes *in vivo*, or monitor whole animals individually or in groups *in situ*. Computer vision is a technology rapidly being implemented in livestock to monitor a host of phenotypes from animal health to efficiency traits (for examples, see: Nasirahmadi et al., 2017). Imaging data can be applied to a wide range of traits noninvasively and inexpensively. These technologies could be used to assess a large number of different phenotypes including some that were not originally considered, such as unique behavioral phenotypes that cannot be measured easily in other ways (Matthews et al., 2017). Wearable sensors can monitor an extensive array of biological processes related to health and traits, such as estrous detection, body temperature, and lameness (Neethirajan, 2017). Animal scientists need to look for ways to apply tools developed for the human health sciences (e.g., Zheng et al., 2014) and plant sciences (Fahlgren et al., 2015) to create diagnostic tools to observe biomarkers that may be useful to determine metabolic state, micronutrient availability, or exposure to infectious disease. Sampling of intestinal microbiomes or other biome niches in the body may also be attainable with internal boluses that can be swallowed (Mimee et al., 2018). An additional important aspect of automated phenotyping is automated collection of management or environmental data at the location of data collection. These data collection technologies may include stationary devices mounted in barns, feeding, or watering troughs that can capture detailed information. Aerial drones have great potential for covering many animals quickly and inexpensively in diverse locations.

Merging data generated on different time scales presents some challenges, but it already is a part of systems such as the US National Dairy Database. For example, birth date, parentage, and SNP genotype do not generally change during an animal’s lifetime (barring errors in recording). Alternatively, lactations tied to a calving event usually span a period of approximately 10 months, test-day samples are taken on a monthly basis, milk weights are typically generated two or three times a day, and activity data from pedometers are generated much more frequently than that. The most formidable challenge in data integration across timescales is ensuring that animal IDs are consistently and correctly reported by disparate systems, and that sampling dates and times are recorded properly.

## Data

### Data Collection Opportunities: Deep Phenotyping and Phenomics

With the advent of high-throughput technologies, the concept of “deep phenotyping” has emerged, providing a more complete picture of phenotypic detail than was previously possible at a specific physiological state (Lanktree et al., 2010; Delude, 2015). Quantitative measures are the emphasis of deep phenotyping because they better differentiate between animals and result in more powerful statistical comparisons than do qualitative measures (i.e., yes/no, grading scales, etc.) (Lanktree et al., 2010). Deep phenotyping of an animal allows for a comprehensive and thorough description of the individual’s physical state at that point in time. The complete description of all of an animal’s phenotypes has been termed its *phenome* (Houle et al., 2010). Deep phenotyping across a group of animals allows for a complete definition of a trait or disease (Robinson, 2012; Delude, 2015). Ideally, deep phenotypes would also include longitudinal data giving an understanding of trait or disease progression/pathophysiology (Haring and Wallaschofski, 2012). Deep phenotyping also allows for separation of different traits that appear similar or identical when measured at the whole-animal level, which facilitates sub-classification of traits and diseases. If a heterogeneous group of animals can be separated into appropriate sub-types, identification of the underlying genetic alleles should be straight forward. An excellent example is the broad classification of respiratory diseases in humans and animals, which could be dissected into specific sub-classes using various microbial sequencing techniques (Langelier et al., 2018).

Disease phenotypes can be sub-classified *a priori* by the presence or absence of particular risk factors or purely on clinical and/or molecular phenotypes (Boland et al., 2013). Environmental risk factors may include exposure to particular endocrine disrupting chemical(s), other pollutants, infectious disease agent(s), a patient’s enterotype (based on gut microbiome bacterial genus and species), or other species present in microbiomes of various mucosa or other niches in the body (Arumugam et al., 2011). Qualitative and quantitative data for disease sub-typing include information from structured data (e.g., laboratory values) and unstructured data (e.g., physical examination, signalment, results of imaging studies, response to specific interventions) (Hripcsak and Albers, 2013). Longitudinal data can be used to quantify changes in traits over time. Animals can be classified into sub-types using supervised or unsupervised clustering machine learning methods (Hripcsak and Albers, 2013). Further sub-classification can be based on molecular phenotypes, including transcriptomic, epigenetic, metagenomic, metabolomic, and proteomic data (Hamada et al., 2016). Ultimately, traits are best sub-classified based on integrative analysis across both physical, clinical and molecular features. Integration of these heterogeneous data (continuous and categorical measures) and classifying based on physiology/pathophysiology is referred to as *endotyping* (Saria and Goldenberg, 2015). In contrast to the endotype, a trait or disease-centric classification, or verotype, is the unique combination of genotypes and phenotypes within a single individual.

### Opportunities for Biomarker Development as High-Throughput Phenotypes

Deep phenotyping aims to improve understanding of physiology and disease pathophysiology. Biomarkers can be developed from deep phenotyping that are sensitive, specific, and relatively inexpensive to detect the trait or disease phenotype of interest for accurate classification of animals. A biomarker is any substance or process that can be measured in a biological specimen that is constantly correlated to the trait of interest (Trusheim et al., 2007). Biomarkers are molecules such as RNA, metabolites, microbes, or proteins/peptides, but may be based on other modalities such as imaging (Hartwell et al., 2006). Ideally, biomarker assays should be minimally invasive, i.e., measurable in peripheral blood or urine (Hartwell et al., 2006; Ziegler et al., 2012). Biomarkers are also used as screening tests for subclinical, asymptomatic, and early-stage diseases. Further, the benefits of early intervention/prevention (including better disease outcomes) should outweigh the costs of performing the screening test (Ziegler et al., 2012). Mid-infrared spectral data can be obtained rapidly and at low cost from samples collected as part of monthly dairy herd improvement programs and there is a growing body of literature which suggests that such data have great potential for predicting risk of disease (Grelet et al., 2016), greenhouse gas emissions (Dehareng et al., 2012), and many other physiological states in the cow (De Marchi et al., 2014; Gengler et al., 2016).

### Defining Big Data in Livestock: How Much Data Are Available in Livestock?

Systematically collected data available for genetic evaluations are among the most extensive in livestock, and are an invaluable tool in genetic progress and understanding physiology and management of animals. Among livestock species, data collected in dairy cattle are among the most numerous and easiest to find in the public setting. However, large amounts of beef cattle, swine, and poultry data also exist for use by genetics companies, but are largely kept proprietary. In 2008, when the first genomic evaluations for US dairy cattle were published, the National Dairy Database (NDDB; Council on Dairy Cattle Breeding, Bowie, MD) contained ∼1,500 sets of 50k bull genotypes (i.e., ∼50,000 SNP genotypes per animal). As of March 13, 2018, it included 2,410,699 genotypes, 2,072,977 of which are cow genotypes. This continual accumulation of genotype data reflected the ability of dairy producers to increase selection in female animals and make rapid genetic gains with genomics (Garcia-Ruiz et al., 2016). **Table 1** shows counts for other types of record in the NDDB.

**Table 1:** Observations in the US national dairy database.

Type of observation	Record count
Pedigree records	75,538,654
Animal genotypes	2,410,699
Lactation records (since 1960)	139,134,191
Daily yield records (since 1990)	684,182,260
Reproduction event records	196,505,574
Health event records	2,541,411
Calving difficulty scores	27,991,336
Stillbirth scores	18,470,886

The rate at which data are being added to livestock databases is rapidly increasing, and the increased frequency of sampling (e.g., from pedometers, rumen boluses, and digital imaging systems) and high dimensionality of some data (e.g., whole-genome DNA sequence and mid-infrared milk spectral data) are driving increases in data transmission and storage requirements. While storage is relatively inexpensive, data transmission is a challenge as many farms do not have access to broadband Internet. This challenge is important because integrated databases containing carefully curated data collected over many years were essential for the implementation of genomic selection (e.g., Wiggans et al., 2017), and will certainly be needed to develop new predictive systems that must be trained on high-quality data. The existing data and rapidly expanding, serially collected data sets are invaluable resources for development of such new predictive analytics.

### Properties of Data

Data collected for use in management and research represent observations of specific phenotypes. Some general principles should be considered when preparing data collection plans. The first of these is that new data should add new information. The ideal case is when new observations have low phenotypic and genetic correlations with existing observations, because new information is being generated. Collection of data that has high correlations with existing data may not be a good use of resources unless the cost of collecting new data is much lower or the new data have lower measurement errors. In cases where data are being collected on commercial farms, there also needs to be a value proposition for the farmer. Ideally, the cost of measurement will be low and the value of the data high.

#### Frequency of Recording

Some observations represent intrinsic (i.e., static) properties of an animal, such as its breed, sex, or genotype. Such data need to be recorded only once in the life of the animal and, in the case of the genotype, may be applied in many different ways. There are other dynamic measurements, such as carcass quality, that can only be recorded one time. In other cases, such as test-day milk yield or daily egg production, phenotypes can be measured many times in an animal’s life. In some cases, additional information can be derived from a sample, such as mid-infrared (MIR) spectra that can be derived from milk samples after initial determination of protein and fat content. Such derived data may have higher dimensionality than the original data, meaning that more information must be transmitted and stored.

The frequency with which data are recorded should be considered when designing data collection and storage systems, with the goal of balancing sampling frequency with predictive accuracy. Storage is not a limiting cost as is it once was, but recording excessive data presents problems when it is time to use them. For example, on-farm weather stations can record temperature, wind speed and direction, barometric pressure, and humidity every minute, but summary statistics for each 24-h period are probably all that is needed to capture important variation. Similarly, digital image data for tracking changes in body condition scores do not need to be captured every time a cow moves across a pen for that purpose, but might be needed to assess changes in activity. Thus, some imaging data may be condensed to fewer observations to acquire the true data of interest (body condition), while more serial collection of other traits (activity) may provide information that is specific to a given period of data based on expected feeding, reproductive, or social behavior. Traditionally, milk samples have been collected and tested on a monthly basis, but this is probably too infrequent for capturing near-real-time changes in fat:protein ratios that are useful for health monitoring, or changes in somatic cell score patterns indicative of subclinical mastitis. Identification of optimal sampling periods is an area that needs active attention from the research community.

#### Dimensionality

The dimensionality of data is, simply, the number of measurements included in an observation; for example, an individual weaning weight or a single test-day milk yield. In most animal production systems, routinely collected data have generally been of low dimension, and there is a close correspondence of the measurement recorded and the phenotype to be measured. The data are generally easy to transmit and store. However, new technologies, such as MIR spectral data, produce many more points of data per observation (hundreds per test-day milk sample), which shows great promise as a low-cost, high-throughput approach for measuring many new phenotypes, particularly those related to milk composition and animal metabolism (e.g., Gengler et al., 2016). There is often a disconnect between the observation (e.g., wavelengths in the spectrum) and the phenotype of interest. Modeling of the phenotype from the observation is more complex, and more resources are needed for data transmission and storage. There are many statistical techniques that can be used to reduce the dimensionality of the data for analysis [e.g., Yao et al. (2012)], but those techniques do not reduce the resources required for transmission or storage of the data.

#### Lifecycle of Data

**Figure 1:**
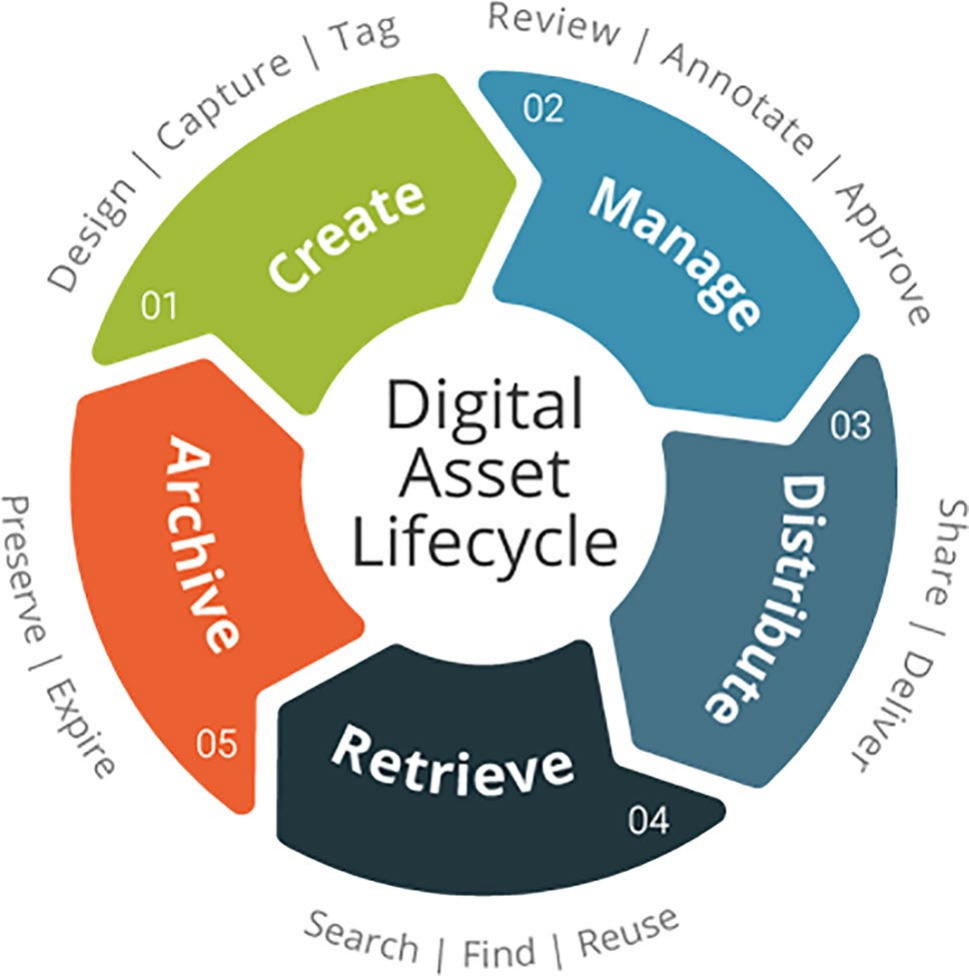
Five-stage data lifecycle model (https://commons.wikimedia.org/wiki/File:Digital-asset-lifecycle.png).

Data, like any other asset, have a lifecycle of their own (**Figure 1**). This five-step model describes each step in the data lifecycle: creation, management, distribution, retrieval, and archiving. In the past, most research efforts focused on creation and management of data because those steps are essential to the scientific enterprise. Distribution is receiving more attention because it is a key step in Open Science initiatives. The rapid growth of large genomics datasets in livestock has been accompanied with greater interest in retrieval, as the popularity of repositories such as the European Variant Archive (Cook et al., 2016) have shown. The final step in the lifecycle, archiving, probably does not receive the attention that it should. Tracking of data creation, management, edits, metadata, and all other aspects of data history are known as data provenance. It takes resources to provide for long-term storage of properly annotated data that is properly replicated, to avoid corruption or loss, and funding for those resources can be difficult to obtain. This is slowly changing as funding agencies place more emphasis on data management plans to ensure that public investments in data are protected, but archiving remains a challenge for individual farmers.

### Curation And Management Of Data

Collections of data have a natural hierarchical structure that can be leveraged to improve organization and annotation. Observations of specific phenotypes for individual animals are at the lowest level. The next level can be a time period of biological significance (such as a lactation) in an animal’s life, or the animal itself (in the case of a terminal production system). Animals are then grouped together to represent different herds or populations which may be separated by time, distance, or both. Finally, those populations can be aggregated into larger collections. The shaded rectangle labeled “Metadata” should include information about the data collected in the next level down. In the case of a herd, for example, metadata would include information such as the herd location, time period sampled, and person responsible for aggregating the data. Additional information may be attached as needed, for example, copies of material license agreements granting permission to possess and use the data for specific digital object identifier (DOI) for manuscripts related to the data, licenses, or standards describing how the phenotypes were defined and collected.

#### Metadata, Ontologies, Thesauri, and Data Dictionaries

In order to make the best use of data, it is important that users of the information have a clear understanding of the content of, structure in, and relationships among the data. Metadata refers to “data about the data,” and can include such information as sources of data, dates of collection, methods used, etc. These data qualifiers, or “tags” provide a deeper, more contextual representation than the data points alone, especially when research data from various researchers is collected and shared in a data repository. Quality metadata that also captures the relationships between tagged data concepts can enhance search and discovery *via* search engines, which may facilitate data assessment and aggregation data across varied research projects or research foci. This can also enable the ability to add recommendations of related materials within a repository to search results. Ontologies, such as the Livestock Product Trait Ontology (https://www.animalgenome.org/bioinfo/projects/lpt/), are curated vocabularies that can be used to ensure that consistent definitions are used to describe phenotypes. The International Committee for Animal Recording (ICAR) also develops international standards for data recording in some livestock species, such as dairy cow health traits [e.g., ICAR Functional Traits Working Group, 2014 (ICAR Functional Traits Working Group, 2014)]. The use of clearly defined terms is strongly recommended to ensure that observations are comparable across datasets. Thesauri such as the National Agricultural Library Thesaurus (https://agclass.nal.usda.gov/) are similar to ontologies and include hierarchical concepts and relationships that can represent the data in context to broader or narrower related concepts. Data dictionaries include definitions of the fields in the data, a description of the relationships among the fields, and the format of the data. Additional details may include permissible ranges/values of observations, data sources, etc. There is clearly some overlap among these types of controlled vocabularies, but the important point is that users of data are provided with comprehensive descriptions of the data, stored as consistent metadata across datasets that may facilitate data-driven discovery and knowledge exchange, and enhance and advance PLF research and use.

#### Data Edits

There is general agreement that quality control (QC) standards are needed to ensure that data being used for analysis are plausible. The basis of any QC system is robust animal identification; without that, it can be difficult or impossible to guarantee that the correct observations are associated with the correct animal. Genotype tests can be useful for identifying and correcting pedigree errors, but the genotype information cannot tell you if a milk weight is assigned to the correct animal or not. Unique ID numbers should be used for each animal whenever possible, while short IDs that are non-unique across locations (sometimes called control numbers) should not be used. The ICAR protocols provides comprehensive guidelines for, and certifies, animal ID devices. The use of ICAR-certified ear tags is recommended whenever possible to ensure that animals are uniquely identified.

The United States dairy sector has developed very sophisticated database-backed systems for processing and storing phenotype, pedigree, and genomic information (Norman et al., 1994; Wiggans et al., 2017). These systems include rules to identify plausible data and ensure consistency across different sources of data. When potentially incorrect data are identified, a complex series of heuristics is used to correct errors whenever possible. The operative philosophy has been to preserve data at all costs, but such a strategy may not be feasible in all cases, particularly as data sources and volume increase. Most research projects will not use such a complex system, but experience has shown that details of data edits are often poorly described in manuscripts. There appears to be general agreement in the literature on QC parameters for SNP genotype date (e.g., call rates, minor allele frequencies, departures from Hardy-Weinberg equilibrium), but there is less agreement when it comes to other types of information. This can be addressed in part by scientists making their software programs available (Ince et al., 2012) using GitHub, or similar services. Tools such as Jupyter Interactive Notebooks (Ragan-Kelley et al., 2014) are very well-suited to this purpose.

#### Repositories

Data are most useful when they can be easily accessed and combined with other information in ways not necessarily anticipated when they were compiled. This is best accomplished by storing data in a repository that others can access freely, and the Human Genome Project showed that the open sharing of data does not inhibit the ability of scientists to make discoveries using open data (Green et al., 2015). As datasets continue to grow in number, there is also a need for a persistent identification scheme that uniquely identifies datasets, including revisions of datasets. The concept of persistent identifiers, such as those provided by the Document Object Identifier (DOI) System, is familiar to most scientists, and the ORCID IDs used by many journals to identify authors can be thought of as a DOI for scientists, rather than publications or datasets. ORCID is a non-profit organization that tracks researcher scholarship, providing unique digital identification numbers to identify researchers and other research outputs (https://orcid.org/).

Data repositories offer a solution to both of these problems, and there are a number of groups that provide general, as well as domain-specific, repositories for datasets. Some repositories are free to use (e.g., Open Science Framework), while others require the payment of a fee (e.g., Dryad Digital Repository). Many repositories also restrict the size of datasets that can be deposited. One curated list of such repositories is provided by the journal Scientific Data (https://www.nature.com/sdata/policies/repositories).

#### Data Preservation

Many individuals have experienced the heartbreak of learning that there was only one copy of important data only after information has been lost. Data preservation is an important, but sometimes tedious, subject. The quote “There is no cloud, there’s just somebody else’s computer” is ubiquitous on the Internet, and while it is intended to skewer the concept of the cloud as a magical solution to all problems, there is an important truth at the heart of it: when you put your data in the cloud, you don’t really know where it is.

### Sharing, Ownership, and Control of Data

The legal issues associated with ownership and control of data are complex, and beyond the scope of this paper. However, a few general points can be discussed here. Large livestock datasets generally are the result of aggregating small datasets from hundreds or thousands of farms, sometimes even across countries (i.e., Interbull genetic evaluations: https://interbull.org/index). It is important to many data providers that their information is protected from unwanted third-party access, which may include regulators, activists, business partners, business competitors, and commodity traders. In a legal context, the term “ownership” has a specific meaning that may not apply in the case of an intangible item, such as data. It may make more sense to think in terms of control of the data, rather than ownership. The use of licensing agreements may provide the control that data creators desire, but at the cost of additional administrative burden on researchers. There are additional issues, such as the ability, or lack thereof, under software license agreements, to move data out of proprietary systems so that it can be aggregated with other data, that may become increasingly important as the number of sensors on farms increases. In fact, many farmers have been surprised to learn that they must pay additional fees in order to move their data from proprietary, cloud-based systems into their on-farm computer systems, which highlights a discrepancy between those who generate data and those who control it. While farmers may accept that they don't own the software that controls equipment they purchase, they probably will not accept the idea that they do not own the data that is generated by that equipment. These issues are not specific to animal breeding, but affect any discipline in the animal sciences in which researchers are permitted access to data that they do not own. Challenges in accessing data from proprietary systems already slow the speed of research. It is difficult to find public funding for the development of software resources that may help integrate, share or summarize data from existing sensor systems for research; however, the development of open software by academics would help to realize the ability to share information to develop better predictive tools (i.e., The Virtual Dairy Brain: https://research.wisc.edu/funding/uw2020/round-3-projects/a-virtual-dairy-farm-brain/).

In the US dairy sector, most data are owned by farmers and breeding companies, not research institutions. Data are often provided to researchers under material license agreements that do not permit use of the data beyond the scope of an individual project, prohibit redistribution of the data, and do not allow deposition of those records in public databases. This allows the industry to retain control of the data while also benefitting from research using those data. These restrictions are a challenge to the research community because, under Open Science initiatives, e.g., (Nosek et al., 2015), a growing number of funding agencies and journals require that the data used in a study be made publicly available. Anonymization has been proposed as one approach to this challenge, but if information can be aggregated from enough sources it is possible to de-anonymize datasets (Wjst, 2010; Bohannon, 2013).

Restrictions imposed by data owners already have had unintended consequences, most notably limiting the ability of scientists to publish in journals that require data deposition. There is general agreement in the scientific community that data should be shared whenever possible, but there is a difference between those who would hoard data for their sole benefit, and those who do not publicly share data because they are not allowed to by the owners of those data. That distinction is not always clearly recognized by data access policies. Some journals have permitted authors to include instructions for readers who would like to request access to the data, although some journals will retract publications if reasonable, good-faith data requests are not granted. If data requirements imposed by publishers do not recognize this challenge then it will have undesirable effects on the ability of many scientists to publish. This will result in either animal breeders focusing most of their effort on topics which do not require access to field data (e.g., computational methods) or on the analysis of data from small trials that cannot readily be generalized to large populations. In the long run, this will limit the ability of animal scientists to solve large-scale problems of substantial importance.

### Users of the Data

**Figure 2:**
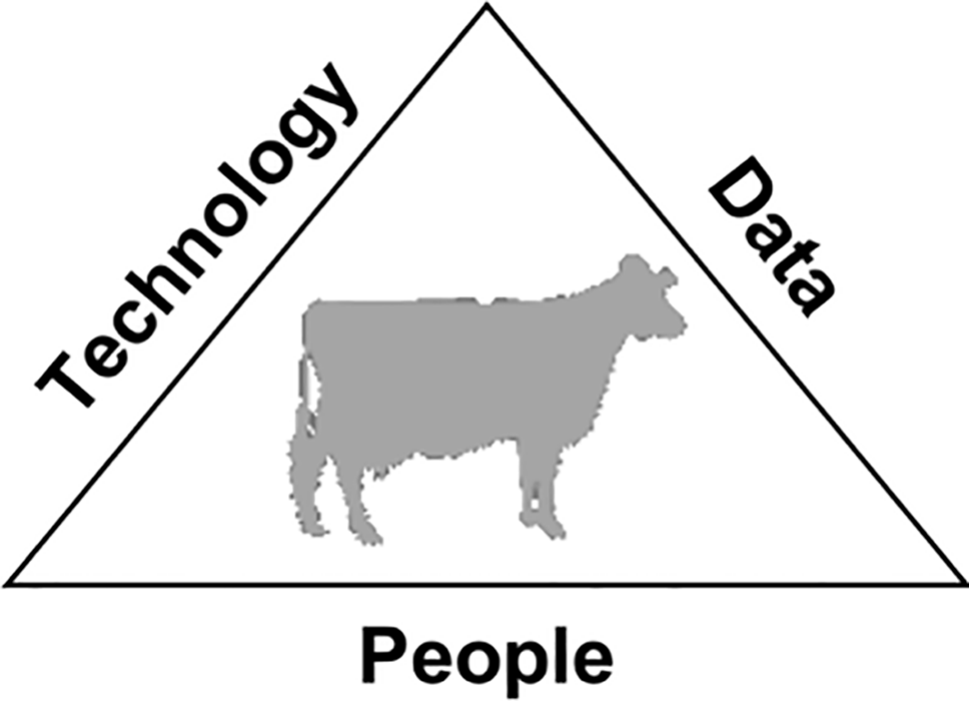
The data-driven decision triad.

There are often multiple users of the same data, each of whom may have different interests. A three-component model showing interactions among people, technology, and data, with livestock at the center, is shown in **Figure 2**. The goal of both data-driven decisions by farmers and commercial entities and data-driven discovery by scientists is to produce nutritious food and fiber for humans using healthy, efficient livestock. Farmers are motivated to collect data because they can be used to make on-farm decisions, ranging from breeding and treatment choices to long-term investment in facilities and equipment. Companies are interested in aggregating data from many different sources to aid in product development and marketing. Scientists are interested in using data to better understand animal biology, both for the sake of knowledge discovery and to help solve problems of production agriculture.

### Recommendations For Data Management

Whenever feasible, the following best practices should be followed:

- Every project should have a feasible data management plan that is followed. Strategies for long-term archiving that support data discovery are critical components of such plans.
- A comprehensive data dictionary defining data types, permissible ranges of values, etc. should accompany each dataset.
- All edits applied to data in an analysis should be reported in detail within a manuscript. Authors are strongly encouraged to use GitHub (or similar resource) to distribute the software programs used for editing data.
- Data should be deposited in public repositories and DOI should be used to associate datasets with publications, and *vice versa*.
- Copies of datasets should be distributed to ensure that they are protected against corruption or loss.

### Animal Phenotypes

It is long-recognized that understanding phenotype, or the display or expression of the genotype in measurable traits, is key to understanding biological systems (Houle et al., 2010) to advance animal agriculture by understanding how to maintain animal health, productivity, and economic sustainability (Hamernik and Adelson, 2003). With the advent of next-generation sequencing (NGS) techniques, the onslaught of genomic sequence data will need to be balanced with increased, high-quality phenotypic data. Detailed, high-quality phenotypes must be generated so we may have a more complete view of the biological information useful for on-farm application or basic scientific knowledge (Lee and Mockler, 2015). Additionally, non-animal factors such as environment or management that can also affect phenotype, become critically important. This is known as the G x E x M, or genome-by-environment-by-management interaction at work to yield particular phenotypes (Hatfield and Walthall, 2015). Starting with a foundation and understanding of existing phenotype collection methods and data, we can integrate these additional non-animal factors, and make the aggregation manageable and fruitful with the development of high-throughput phenotyping technologies, and the data management systems and training needed to manage them.

The United Nations Food and Agriculture Organization (FAO) declared in its *Second Report on the State of the World’s Animal Genetic Resources for Food and Agriculture*, that, despite significant advances in genomics, the impact of this progress is muted due to limitations in phenotyping and pedigree data, and that, “Increasing the collection of these data is of critical importance, not only for the effective use of genomics, but for any type of genetic improvement of conservation program.” (The Second Report on the State of the World’s Animal Genetic Resources for Food and Agriculture, 2015) Animal phenotypes represent a number of biological systems, layered and integrated with each other, that are all informed by, or reflected in their associated animal genotypes. Thus, animal phenotypes include a wide range of both complexity, and collection methods, from blood chemistries to body weights, from individual animal behaviors to group dynamics, and population adaptations and migrations. Additionally, data regarding production systems, geography and climate, and socio-economic factors that may influence phenotypes, add additional layers, sources, formats, and complexity of data. Animal phenotypes traditionally have been collected often simply, but in time consuming ways one animal at a time. For example, one blood draw, one run through the scale, or two weights of feed are measured—one before, and another after the meal for each animal to determine feed intake. To collect data on a population, this is repeated as many times as money and time allows. Today, records from these types of sampling may still be collected with pencil and paper for later transfer to computer, or directly onto a handheld device in a digital format. There have been advances, for example, researchers can now install feed bins that can register an animal ID on its collar as it comes up to feed, and weights are taken automatically every few seconds and sent automatically to a computer for analyses to monitor feed intake. Other sampling techniques remain largely unchanged, such as the blood draw to measure a vast number of phenotypes seen in blood analyses, which may as an example, inform animal health and productivity in the field of metabolomics (Rexroad et al., 2019).

## Summary

Big data has been common place in livestock genetic evaluations for decades, but new technologies have created the ability to automatically collect phenotypes on unprecedented scales. The first wave of big data came with molecular assays such as genotyping and sequencing, but sensors, imaging systems and other devices have created the ability to continuously phenotype animals at unprecedented levels. Though many aspects related to genetics have been presented in this study, all disciplines within animal science will benefit and grow with the continued development of these high-throughput technologies. Defined challenges of big data in livestock include how to share data across institutes and private entities, how to standardize data recording, management, QC, trait terminology, and data processing. Innovative approaches will be needed to differentiate information in data from noise, develop accurate prediction models, and integrate information across diverse sources and locations. Opportunities to leverage crowd-sourcing, machine learning based artificial intelligence and other innovative data transfer and storage approaches will be critical to glean knowledge from livestock data. Many data, such as those generated in the dairy industry in milk testing, are already available for immediate use. Utilization, reuse, and generation of data has great potential to enhance the efficiency, welfare, and societal benefit of livestock. Deep phenotyping also has the potential to provide great benefits to society by providing detailed basic physiological knowledge that cannot be measured on humans or model organisms on a routine basis. Training people to work with big data will provide tremendous opportunities for academia and private industry to develop knowledge and tools to sustainably feed a growing world.

## Author Contributions

All authors participated in the meeting and helped write the paper.

## Funding

This meeting was supported by a competitive USDA-NIFA grant # 2017-67015-26907.

## Conflict of Interest

The authors declare that the research was conducted in the absence of any commercial or financial relationships that could be construed as a potential conflict of interest.

The reviewer LB declared a past co-authorship with one of the authors JC to the handling editor.

## References

[B1] ACM (2011). CERN Experiments Generating One Petabyte of Data Every Second. http://cacm.acm.org/news/110048-cern-experiments-generating-one-petabyte-ofdata-every-second/fulltext.

[B2] Arumugam M. Raes J. Pelletier E. Le Paslier D. Yamada T. Mende D. R. (2011). Enterotypes of the human gut microbiome. Nature 12 (4737346), 174–180. 10.1038/nature09944 PMC3728647 21508958

[B3] Berckmans D. (2017). General introduction to precision livestock farming. Anim. Front. 7 (1), 6–11. 10.2527/af.2017.0102

[B4] Bohannon J. (2013). Genetics. Genealogy databases enable naming of anonymous DNA donors. Sci. (New York N.Y.) 339 (6117), 262. 10.1126/science.339.6117.262 23329025

[B5] Boland M. R. Hripcsak G. Shen Y. Chung W. K. Weng C. (2013). Defining a comprehensive verotype using electronic health records for personalized medicine. J. Am. Med. Inf. Assoc.: JAMIA 20 (e2), e232–e238. 10.1136/amiajnl-2013-001932 PMC3861934 24001516

[B6] Cole J. B. Newman S. Foertter F. Aguilar I. Coffey M. Wiggans G. (2012). Breeding and genetics symposium: really big data: processing and analysis of very large data sets. J. Anim. Sci. 90, 723–733. 10.2527/jas.2011-4584 22100598

[B7] Cook C. E. Bergman M. T. Finn R. D. Cochrane G. Birney E. Apweiler R. (2016). The European Bioinformatics Institute in 2016: data growth and integration. Nucleic Acids Res. 2015 (44), D20–D26. 10.1093/nar/gkv1352 PMC4702932 26673705

[B8] Daetwyler H. D. Capitan A. Pausch H. Stothard P. van Binsbergen R. Brøndum R. F. (2014). Whole-genome sequencing of 234 bulls facilitates mapping of monogenic and complex traits in cattle. Nat. Genet. 46 (8), 858–865. 10.1038/ng.3034 25017103

[B9] de Beer J. (2016). Ownership of Open Data: Governance Options for Agriculture and Nutrition. Global Open Data for Agriculture & Nutrition (GODAN) Report. (Oxfordshire, United Kingdom: Nosworthy Way Wallingford). 10.1079/CABICOMM-79-13

[B10] De Marchi M. D. Toffanin V. Cassandro M. Penasa M. (2014). Invited review: min-infrared spectroscopy as phenotyping tool for milk traits. J. Dairy Sci. 97 (3), 1171–1186. 10.3168/jds.2013-6799 24440251

[B11] Dehareng F. Delfosse C. Froidmont E. Soyeurt H. (2012). Potential use of milk mid-infrared spectra to predict individual methane emission of dairy cows. Animal 6, 1694–1701. 10.1017/S1751731112000456 23031566

[B12] Delude C. M. (2015). Deep phenotyping: the details of disease. Nature 527 (7576), S14–S15. 10.1038/527S14a 26536218

[B13] Dorea J. R. R. Rosa G. J. M. Weld K. A. Armentano L. E. (2018). Mining data from milk infrared spectroscopy to improve feed intake predictions in lactating dairy cows. J. Dairy Sci. 101 (7), 5878–5889. 10.3168/jds.2017-13997 29680644

[B14] Employment Opportunities for College Graduates. (2015). https://www.purdue.edu/usda/employment/wp-content/uploads/2015/04/2-Page-USDA-Employ.pdf.

[B15] Fahlgren N. Gehan M. A. Baxter I. (2015). Lights, camera, action: high-throughput plant phenotyping is ready for a close-up. Curr. Opin. Plant Biol. 24, 93–99. 10.1016/j.pbi.2015.02.006 25733069

[B16] Food and Agriculture Organization of the United Nations (FAO) (2018). Shaping the Future of Livestock. Berlin: The 10th Global Forum for Food and Agrculture, http://www.fao.org/3/i8384en/I8384EN.pdf.

[B17] Garcia-Ruiz A. Cole J. B. VanRaden P. M. Wiggans G. R. Ruiz-Lopez F. J. Van Tassell C. P. (2016). Changes in genetic selection differentials and genreation intervals in US Holstein dairy cattle as a result of genomic selection. Proc. Natl. Acad. Sci. U. S. A. 113 (28), E3995–E4004. 10.1073/pnas.1519061113 27354521 PMC4948329

[B18] Gengler N. Soyeurt H. Dehareng F. Bastin C. Colinet F. Hammami H. (2016). Capitalizing on fine milk composition for breeding and management of dairy cows. J. Dairy Sci. 99, 4071–4079. 10.3168/jds.2015-10140 26778306

[B19] Green E. D. Watson J. D. Collins F. S. (2015). Human genome project: twenty-five years of big biology. Nature 526 (7571), 29–31. 10.1038/526029a 26432225 PMC5101944

[B20] Grelet C. Bastin C. Gelé M. Davière J. B. Johan M. Werner A. (2016). Development of Fourier transform mid-infrared calibrations to predict acetone, β-hydroxybutyrate, and citrate contents in bovine milk through a European dairy network. J. Dairy Sci. 99, 4816–4825. 10.3168/jds.2015-10477 27016835

[B21] Guarino M. Norton T. Berckmans D. Vranken E. Berckmans D. (2017). A blueprint for developing and applying precision livestock farming tools: a key output of the EU-PLF project. Anim. Front. 7 (1), 12–17. 10.2527/af.2017.0103

[B22] Hamada T. Keum N. N. Nishihara R. Ogino S. (2016). Molecular pathological epidemiology: new developing frontiers of big data science to study etiologies and pathogenesis. J. Gastroenterol. 52 (3), 1–11. 10.1007/s00535-016-1272-3 27738762 PMC5325774

[B23] Hamernik D. L. Adelson D. L. (2003). USDA stakeholder workshop on animal bioinformatics: summary and recommendations. Comp. Funct. Genomics 4 (2), 271–274. 10.1002/cfg.266 18629125 PMC2447412

[B24] Haring R. Wallaschofski H. (2012). Diving through the ‘-Omics’: the case for deep phenotyping and systems epidemiology. Omics 16 (5), 231–234. 10.1089/omi.2011.0108 22320900 PMC3339382

[B25] Hartwell L. Mankoff D. Paulovich A. Ramsey S. Swisher E. (2006). Cancer biomarkers: a systems approach. Nat. Biotechnol. 24 (8), 905–908. 10.1038/nbt0806-905 16900126

[B26] Hatfield J. L. Walthall C. L. (2015). Meeting global food needs: realizing the potential *via* Genetics × Environment × Management Interactions. Agron. J. 107 (4), 1215–1226. 10.2134/agronj15.0076

[B27] Hill W. G. (2016). Is continued genetic improvmnt of livestock sustainable. Genetics 202, 877–881. 10.1534/genetics.115.186650 26953266 PMC4788124

[B28] Houle D. Govindaraju D. R. Omholt S. (2010). Phenomics: the next challenge. Nat. Rev. Genet. 11 (12), 855–866. 10.1038/nrg2897 21085204

[B29] Hripcsak G. Albers D. J. (2013). Next-generation phenotyping of electronic health records. J. Am. Med. Inf. Assoc.: JAMIA 20 (1), 117–121. 10.1136/amiajnl-2012-001145 PMC3555337 22955496

[B30] ICAR Functional Traits Working Group (2014). “Section 7.1 – guidelines for recording, evaluation and genetic improvement of health traits,” in ICAR Recording Guidelines (Rome, Italy: ICAR), 235–261. http://citeseerx.ist.psu.edu/viewdoc/download?doi=10.1.1.664.1466&rep=rep1&type=pdf.

[B31] Ince D. C. Hatton L. Graham-Cumming J. (2012). The case for open computer programs. Nature 482 (7386), 485–488. 10.1038/nature10836 22358837

[B32] Kuehne G. Llewellyn R. Pannell D. J. Wilkinson R. Dolling P. Ouzman J. (2017). Predicting farmer update of new agricultural practices: a tool for research, extension and policy. Agric. Syst. 156, 115–125. 10.1016/j.agsy.2017.06.007

[B33] Langelier C. Kalantar K. L. Moazed F. Wilson M. R. Crawford E. D. Deiss T. (2018). Integrating host response and unbiased microbe detection for lower respiratory tract infection diagnosis in critically ill adults. Proc. Natl. Acad. Sci. U. S. A. 115 (52), E12353–E12362. 10.1073/pnas.1809700115 30482864 PMC6310811

[B34] Lanktree M. B. Hassell R. G. Lahiry P. Hegele R. A. (2010). Phenomics: expanding the role of clinical evaluation in genomic studies. J. Invest. Med.: Off. Publ. Am. Fed. Clin. Res. 58 (5), 700–706. 10.231/JIM.0b013e3181d844f7 20216460

[B35] Lee I. Mockler T. C. (2015). Editorial overview: genome studies and molecular genetics: data-driven approaches to genotype-to-phenotype studies in crops. Curr. Opin. Plant Biol. 24 (April), iv–vi. 10.1016/j.pbi.2015.03.001 25817324

[B36] Matthews S. G. Miller A. L. Plotz T. Kyriazakis I. (2017). Automated tracking to measure behavioural changes in pigs for health and welfare monitoring. Sci. Rep. 7, 17582. 10.1038/s41598-017-17451-6 29242594 PMC5730557

[B37] McCue M. E. McCoy A. M. (2017). The scope of big data in one medicine: unprecedented opportunities and challenges. Front. Vet. Sci. 4, 194. 10.3389/fvets.2017.00194 29201868 PMC5696324

[B38] Mimee M. Nadeau P. Hayward A. Carim S. Flanagan S. Jerger L. (2018). An ingestible bactrial-electronic system to monitor gastrointestinal health. Science 360 (6391), 915–918. 10.1126/science.aas9315 29798884 PMC6430580

[B39] Morota G. Ventura R. V. Silva F. F. Koyama M. Fernando S. C. (2018). BIG data analytics and precision animal agricukture symposium: machine learning and data mining advance predictive big data analysis in precision animal agriculture. J. Anim. Sci. 96, 1540–1550. 10.1093/jas/sky014 29385611 PMC6140937

[B40] Nasirahmadi A. Edwards S. A. Sturm B. (2017). Implementation of machine vision for detecting behaviour of cattle and pigs. Livestock Sci. 202, 25–38. 10.1016/j.livsci.2017.05.014

[B41] Neethirajan S. (2017). Recent advances in wearable sensors for animal health management. Sens. Bionsensing Res. 12, 15–29. 10.1016/j.sbsr.2016.11.004

[B42] Norman H. D. Waite L. G. Wiggans G. R. Walton L. M. (1994). Improving accuracy of the united states genetics database with a new editing system for dairy records. J. Dairy Sci. 77 (10), 3198–3208. 10.3168/jds.S0022-0302(94)77263-5

[B43] Nosek B. A. Alter G. Banks G. C. Borsboom D. Bowman S. D. Breckler S. J. (2015). SCIENTIFIC STANDARDS. Promoting an open research culture. Sci. (New York N.Y.) 348 (6242), 1422–1425. 10.1126/science.aab2374 PMC4550299 26113702

[B44] Pierpaoli E. Carli G. Pignatti E. Canavari M. (2013). Drivers of precision agriculture technologies adoption: a literature review. Proc. Technol. 8, 61–69. 10.1016/j.protcy.2013.11.010

[B45] Platt J. R. (1964). Strong inference. Science 146 (3642), 347 LP–347353. 10.1126/science.146.3642.347 17739513

[B46] Ragan-Kelley M. Perez F. Granger B. Kluyver T. Ivanov P. Frederic J. (2014). “The Jupyter/IPython architecture: a unified view of computational research, from interactive exploration to communication and publication,” in American Geophysical Union. Fall Meeting 2014, Abstract Id. H44D-07. http://adsabs.harvard.edu/abs/2014AGUFM.H44D.07R.

[B47] Ramirez B. C. Xin H. Halbur P. G. Beerman D. H. Hansen S. L. Linhares D. C. L. (2019). At the intersection of industry, academia and government: How do we facilitate productive precision livestock farming in practice?. Animals 9 (9), E635. 10.3390/ani9090635 31480220 PMC6770712

[B48] Rexroad C. Vallet J. Matukumalli L. K. (2019). Genome to phenome: improving animal health, production, and well-being - a new USDA blueprint for animal genome research 2018-2027. Front. Genet. 10, 327. 10.3389/fgene.2019.00327 31156693 PMC6532451

[B49] Robinson P. N. (2012). Deep phenotyping for precision medicine. Hum. Mutat. 33 (5), 777–780. 10.1002/humu.22080 22504886

[B50] Rutten C. J. Velthuis A. G. J. Steeneveld W. Hogeveen H. (2013). Invited review: sensors to support health management on dairy farms. J. Dairy Sci. 96 (4), 1928–1952. 10.3168/jds.2012-6107 23462176

[B51] Saria S. Goldenberg A. (2015). Subtyping: what it is and its role in precision medicine. IEEE Intell. Syst. 30 (4), 70–75. 10.1109/MIS.2015.60

[B52] Science Breakthroughs to Advance Food and Agricultural Research by 2030 (2019). Committee on Science Breakthroughs 2030: A strategy for Food and Agricultural Research. 500 Fifth Street, NW Washington, DC: The National Academies Press. 10.17226/25059

[B53] Stubbs M. (2016). Big Data in U.S. Agriculture Specialist in Agricultural Conservation and Natural Resources Policy. https://fas.org/sgp/crs/misc/R44331.pdf.

[B54] The Second Report on the State of the World’s Animal Genetic Resources for Food and Agriculture (2015). FAO Commission on Genetic Resources for Food and Agriculture Assessments. Rome: FAO.

[B55] Trusheim M. R. Berndt E. R. Douglas F. L. (2007). Stratified medicine: strategic and economic implications of combining drugs and clinical biomarkers. Nat. Rev. Drug Discovery 6 (4), 287–293. 10.1038/nrd2251 17380152

[B56] USDA Rural Development Innovation Center (2019). e-Connectivity @ USDA: Broadband Resources for Rural America. https://www.rd.usda.gov/files/508_RDeConnectivityToolkit121918.pdf.

[B57] Van Hertem T. Rooijakkers L. Pena Fernandez A. Norton T. Berkmans D. Vranken E. (2017). Appropriate data visualization is key to Precision Livestock Farming acceptance. Comput. Electron. Agric. 138 (1), 1–10. 10.1016/j.compag.2017.04.003

[B58] Weersink A. Fraser E. Pannell D. Duncan E. Rotz S. (2018). Opportunities and challenges for big data in agricultural and environmental analysis. Annu. Rev. Resour. Econ. 10, 19–37. 10.1017/S175173111800246X

[B59] Wiggans G. R. Cole J. B. Hubbard S. M. Sonstegard T. S. (2017). Genomic selection in dairy cattle: the USDA experience. Annu. Rev. Anim. Biosci. 5 (1), 309–327. 10.1146/annurev-animal-021815-111422 27860491

[B60] Wjst M. (2010). Caught you: threats to confidentiality due to the public release of large-scale genetic data sets. BMC Med. Ethics 11 (1), 21. 10.1186/1472-6939-11-21 21190545 PMC3022540

[B61] Wolfert S. Ge L. Verdouw C. Bogaardt M.-J. (2017). Big data in smart farming- a review. Agric. Syst. 153, 69–80. 10.1016/j.agsy.2017.01.023

[B62] Yao F. Coquery J. Cao K.-A. L. (2012). Independent principal component analysis for biologically meaningful dimension reduction of large biological data sets. BMC Bioinf. 13 (1), 24. 10.1186/1471-2105-13-24 PMC3298499 22305354

[B63] Zegura E. Grinter B. Belding E. Nahrstedt K. (2017). A rural lens of a research agenda for intelligent infrastructure. arXiv:1705.02004. https://cra.org/ccc/wp-content/uploads/sites/2/2017/05/ARuralLensonaResearchAgendaforIntelligentInfrastructure-FINAL.pdf.

[B64] Zheng Y.-L. Ding X.-R. Poon C. C. Y. Lo B. P. L. Zhang H. Zhou X.-L. (2014). Unobtrusive sensing and wearable devices for health informatics. IEEE Trans. Biomed. Eng. 61, 1538–1554. 10.1109/TBME.2014.2309951 24759283 PMC7176476

[B65] Ziegler A. Koch A. Krockenberger K. Großhennig A. (2012). Personalized medicine using dna biomarkers: a review. Hum. Genet. 131 (10), 1627–1638. 10.1007/s00439-012-1188-9 22752797 PMC3432208

